# Autoantibodies to Chemokines and Cytokines Participate in the Regulation of Cancer and Autoimmunity

**DOI:** 10.3389/fimmu.2018.00623

**Published:** 2018-03-29

**Authors:** Nathan Karin

**Affiliations:** Department of Immunology, Faculty of Medicine, Technion – Israel Institute of Technology, Haifa, Israel

**Keywords:** autoantibodies, chemokines, cytokines, experimental autoimmune encephalomyelitis, type I diabetes, cancer, tolerance

## Abstract

We have previously shown that predominant expression of key inflammatory cytokines and chemokines at autoimmune sites or tumor sites induces loss of B cells tolerance, resulting in autoantibody production against the dominant cytokine/chemokine that is largely expressed at these sites. These autoantibodies are high-affinity neutralizing antibodies. Based on animal models studies, we suggested that they participate in the regulation of cancer and autoimmunity, albeit at the level of their production cannot entirely prevent the development and progression of these diseases. We have, therefore, named this selective breakdown of tolerance as “Beneficial Autoimmunity.” Despite its beneficial outcome, this process is likely to be stochastic and not directed by a deterministic mechanism, and is likely to be associated with the dominant expression of these inflammatory mediators at sites that are partially immune privileged. A recent study conducted on autoimmune regulator-deficient patients reported that in human this type of breakdown of B cell tolerance is T cell dependent. This explains, in part, why the response is highly restricted, and includes high-affinity antibodies. The current mini-review explores this subject from different complementary perspectives. It also discusses three optional translational aspects: amplification of autoantibody production as a therapeutic approach, development of autoantibody based diagnostic tools, and the use of B cells from donors that produce these autoantibodies for the development of high-affinity human monoclonal antibodies.

## Introduction

Autoantibodies to self-components are commonly associated with the development of autoimmunity, allergic diseases, and scleroderma (1–6). As opposed to these harmful antibodies, here we focus on those that are being produced during pathological conditions and are beneficial for the host. About 14 years ago, we have identified that along the course of rheumatoid arthritis (RA), the immune system produces neutralizing autoantibodies against tumor necrosis factor alpha (TNFα) (7), one of key drivers of the inflammatory process in this disease (8). Similar autoantibodies have also been observed in rodents after the induction of experimentally induced arthritis (i.e., adjuvant-induced arthritis), even before the onset of disease (7). In these rodents, amplification of this response by a targeted DNA vaccine encoding TNFα suppressed the experimental disease, whereas their elimination *via* induction of neonatal tolerance to TNFα aggravated the severity of disease (7). Collectively, this implies for a selective breakdown of B-cell tolerance that restrains destructive autoimmunity. The relevance of such beneficial protective autoantibodies has recently been highlighted in a study focusing on breakdown of B-cell tolerance in APS1/APECED patients (9). These patients have a functional deficiency of the autoimmune regulator (AIRE) gene that is essential for the generation of central T cell tolerance to many self-antigens (10–12). The study showed that in the absence of central T cell tolerance the immune system promoted T-dependent high-affinity autoantibody production to key cytokines, and by so doing provokes resistance to autoimmunity (9). The disease in focus in this manuscript is type I diabetes (T1DM) and autoantibodies that are likely to affect the development of T1DM are produced against type-I interferons (9).

The current mini-review describes how we discovered this type of regulatory response long ago, and its relevance to cancer and autoimmunity. It also discusses the implications of these findings for therapy, diagnosis, and development of therapeutic human monoclonal antibodies.

## The Discovery of Autoantibody-Based Regulation of Autoimmunity and Cancer

Almost 20 years ago, we have applied the DNA vaccination technology to induce anti-chemokine autoantibody production and by so doing explore their differential role in the regulation of autoimmunity (13–16). The basic idea has been to inject rodents with CpG-enriched plasmid DNA encoding different chemokines or cytokines, and then to follow the effect of these vaccines on the generation of neutralizing autoantibodies to chemokines/cytokines and on the development and progression of the autoimmune condition. In continuing experiments, these autoantibodies were purified and their disease protective abilities were confirmed by adoptive transfer experiments (13–16). In one of these experiments, a CpG-enriched plasmid DNA vaccine encoding TNFα was administered just after the onset of experimental autoimmune encephalomyelitis (EAE), and surprisingly, its beneficial effect was very rapid (17). It should be noted that in contrast to EAE in multiple sclerosis, it is not clear whether TNFα suppresses or aggravates the disease (18, 19). Later, we learned that the generation of high antibody titer following administration of targeted DNA vaccines is very rapid because it amplifies an existing autoantibody response that by itself restrains the dynamics of these diseases (7). Finally, we have extended these experiments to cancer, showing that in these diseases anti-chemokine autoantibody production is apparent and could be amplified in a beneficial manner (20). The link between cancer and autoimmunity is that in both types of diseases some chemokines and cytokines are largely expressed at site that are partially segregated from the immune surveillance, as described below.

## Our Working Hypothesis

Why are autoantibodies to inflammatory cytokines and chemokines being selectively produced in cancer and autoimmune diseases?

**Figure 1:**
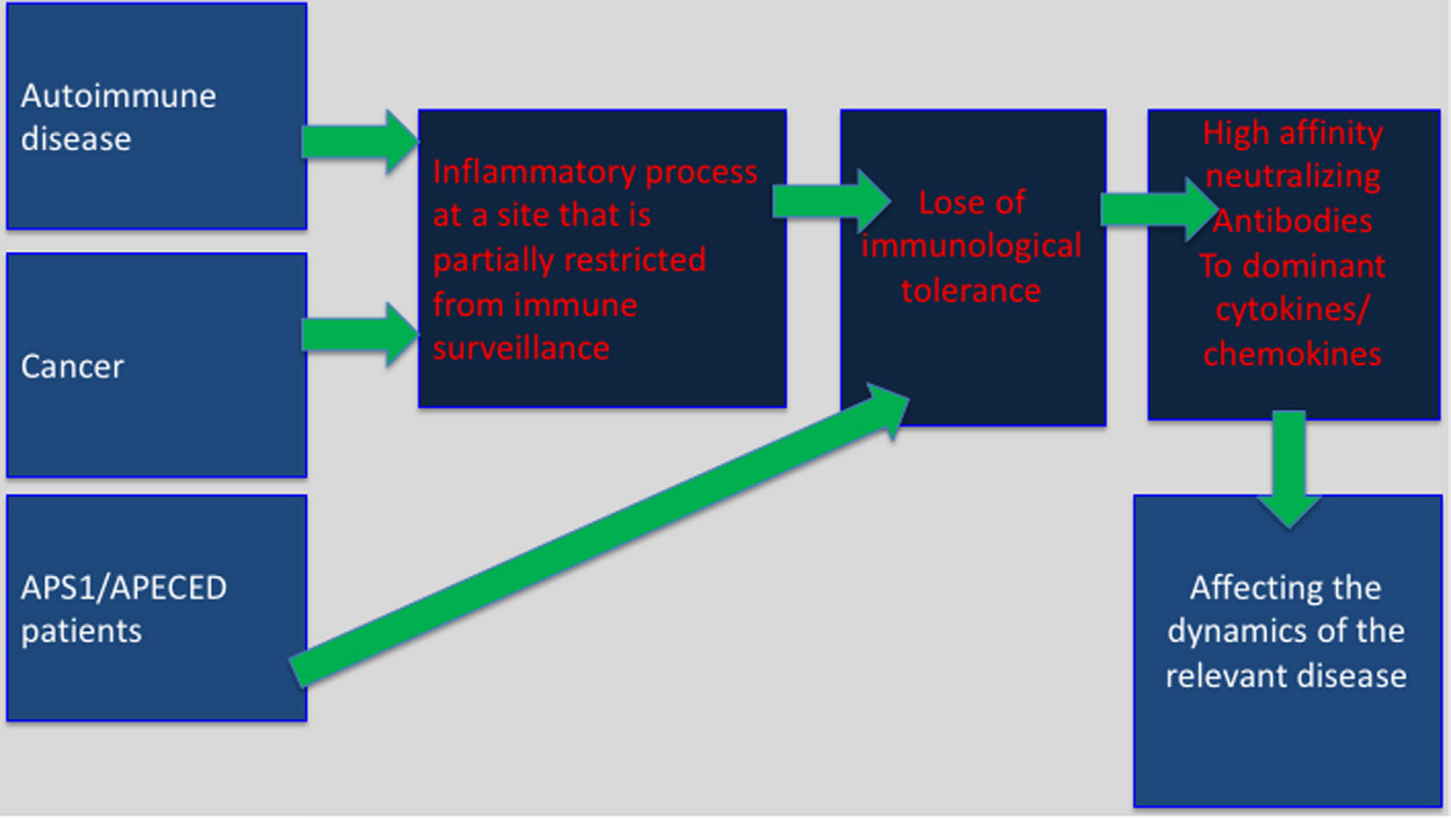
Selective breakdown of B cell tolerance to key inflammatory cytokines/chemokines regulates cancer, autoimmunity and infectious diseases: selective breakdown of tolerance to chemokines and cytokines may result from the generation of an inflammatory process at sites that are partially restricted from immune surveillance (autoimmune sites, tumor sites) or as a results of a deficiency in central tolerance (APS1/APECES patients with autoimmune regulator deficiency). In both, it is believed that breakdown of B cell tolerance follows the breakdown of T cell tolerance. The data obtained from APS1/APECES patients strongly support this hypothesis.

Immune privilege sites were originally believed to be associated with particular organs, which were believed to require superior protection from an excessive inflammatory activity that might cause direct damage to these organs. Key examples are as follows: the testes, brain, the anterior chamber of the eye, and the placenta. It is likely that in these areas the ability of regulatory T cells (T_reg_) to restrain anti-self-immunity, under inflammatory conditions, is limited. This may explain, in part, the development of bystander autoimmunity following an inflammatory process within “classical” immune privileged sites (21). A newer and more comprehensive interpretation of immune privilege sites suggests that they can be acquired locally in many different tissues in response to self antigens (22). Aside of autoimmune sites this may also include tumor sites, and the tumor-draining lymph nodes (23). Our working hypothesis is that in these sites predominant expression of inflammatory cytokines or chemokines may lead to T-dependent breakdown of tolerance resulting in anti-inflammatory cytokines/chemokines autoantibody production (Figure 1).

Although it is tempting to speculate that the generation of these autoantibodies is deterministic, for the benefit of the host, it is more likely that their production is stochastic, and is due to the overexpression of gene products under inflammatory conditions at a site that is partially segregated from immune surveillance. Such conditions may give rise to a selective breakdown of immunological tolerance (24). As a stochastic process, it may also enable the production of autoantibodies that would be harmful to the host, such as anti-SR-A antibodies in systemic lupus erythematosus (25).

## Anti-TNF-α Autoantibodies in RA and Psoriasis

Rheumatoid arthritis is a systemic autoimmune disease of the joints. Many pro-inflammatory cytokines, including TNFα, IL-1 chemokines, and growth factors, are expressed in diseased joints and have been associated with the development of the inflammatory process resulting in the degeneration of cartilage and erosion of juxta-articular bone (26, 27). In many studies, it has been shown that systemic administration of anti-TNFα antibody or soluble TNFα receptor fusion protein holds a beneficial effect for a major portion of the RA patients, implicating for the pivotal role of this cytokine in the pathogenesis of RA (28). Soluble levels of TNFα receptor showed positive correlation with disease activity in Inflammatory Bowel’s disease (29). We identified the appearance of neutralizing autoantibodies to TNFα in the sera of RA patients, but not in sera of those developing osteoarthritis (7). Mapping of the target epitopes they bind revealed three epitopes on TNFα with very low cross reactivity to any known human protein (7). Thus, breakdown of tolerance to TNFα is highly selective and target specific.

Psoriasis is an inflammatory autoimmune disease of the skin. IL-17, TNFα, and IFNα are all key cytokines that promote the development and progression of disease (30–33). IL-17 and his receptor and TNFα are key targets for therapy in this disease (30–33). We could observe a significant titer of anti-TNFα and IFN-α in patients with psoriasis compared to healthy subjects and patients with atopic dermatitis (34). These antibodies were found to be neutralizing antibodies. So were anti-TNFα antibodies in RA patients. We, therefore, think that they may possibly participate in the regulation of each disease; albeit in the low titer of their production cannot fully prevent its development and progression.

## Anti-CCL3 Autoantibodies in Type I Diabetes

Type-1 diabetes mellitus (T1DM) is an organ-specific autoimmune disease resulted from the destruction of the insulin-secreting β-cells in the pancreatic islets of Langerhans (35). In this disease CD4^+^ and CD8^+^ T cells, macrophages and perhaps NK cells are required for β-cell destruction (36). The destruction of insulin-producing β-cells is likely to be directed by auto-reactive T cells that recognize several islet β-cells antigens. Among the well-characterized autoantibodies in this disease are as follows: anti-insulin Abs (CIAA), islet cell Abs (ICA), glutamic acid decarboxylase (GAD) 65 and 67 isotypes Abs, heat shock protein 60, and some uncharacterized β-cells antigens (37–39). Currently, the diagnosis of T1DM is based on measuring autoantibodies to GAD, ICA, ICA 512 (IA-2), and insulin (40–47). We have followed potential development of autoantibody titer to many different inflammatory cytokines and chemokines in the sera of T1DM patients and observed a significant autoantibody titer to a single dominant chemokine: CCL3 (48). Independently, Cameron et al. observed in NOD mice predominant expression of intra-pancreatic CCL3 in NOD mice, and along with this that CCR3 ko NOD mice display high resistance to T1DM (49). These results, together with ours may suggest that preferred expression of an inflammatory cytokine/chemokine at an autoimmune site that undergoes a destructive process may induce breakdown of tolerance and generation of autoantibodies to this predominant mediator, and that for anti-CCL3 it might be beneficial for the host.

## Anti-CCL2 Autoantibodies in Cancer

The tumor microenvironment is the cellular environment in which the tumor exists. In addition to cancer cells, it includes different cells of either hematopoietic origin, or from mesenchymal origin, and also forms non-cellular components, all of which affect tumor development either due to a direct cross-talk with the tumor, or *via* affecting immune cells functions, within the TEM (50). Cells of the hematopoietic origin consists of cells that arise in the bone marrow and can be subdivided into cells of the lymphoid lineage, consisting of T cells, B cells, and natural killer cells, and those of the myeloid lineage, which includes macrophages, neutrophils, and myeloid-derived suppressor cells (MDSCs). Several studies, including ours, showed that the CCR2–CCL2 axis is critical for the mobilization from the BM to the blood, and later to the tumor site of tumor-associated macrophages to support it development and suppress anti-tumor immunity (20, 51–56). Very recently, we uncovered the mechanism by which “neutrophil like” polymorphonuclear MDSCs are mobilized from the bone marrow to the blood to support tumor development (57). We have observed in patients suffering from cancer of the prostate the development of a significant antibody titer of neutralizing antibodies to the CC chemokine CCL2 (20). Similarly, in an immunocompetent model of the disease in mice these antibodies were also apparent, and the amplification of their level by a targeted DNA vaccine encoding CCL2 rapidly suppressed the development and progression of disease (20).

What do autoimmune sites and tumor sites have in common? Autoimmune sites, tumor sites, and the tumor-draining lymph nodes are partially immune previlaged, and in easch inflammatory cytokines/chemokins are largely expressed (23). Our working hypothesis is that in these sites predominant expression of inflammatory cytokines or chemokines may lead to T-dependent breakdown of tolerance resulting in anti-inflammatory cytokines/chemokines autoantibody production (Figure 1).

## Supporting Evidence for T-Dependent Autoantibody Production from AIRE-Deficient Patients

Mapping of the target determinants to which anti-cytokine/chemokine autoantibodies are produced implicates for a highly restricted response (7). The antibodies that are being produced are mostly of the IgG isotype (IgG1 for human and IgG2a for mouse) (7). Together this implies for a possible T-dependent antibody production. Supporting evidence for T-dependent breakdown of tolerance resulting in neutralizing autoantibody production against cytokines that regulate autoimmunity came form a recent study that analyzes autoantibody production in APS1/APECED patients that due to genetic mutation do not express functional patients’ loss of B cell tolerance that appears to be a T-dependent step (9). In the lack of T-dependent central B cell tolerance, these patients display very high-affinity, neutralizing autoantibodies, particularly against specific cytokines. Such antibodies were biologically active *in vitro* and *in vivo* (9). Clear association between the appearance of antibodies to type-1 interferons and T1DM could be observed (9). Based on our previous observations and these findings, we suggest a model in which the expression of inflammatory cytokines at sites that are partially segregated from immune surveillance would induce T-dependent loss of B cell tolerance and generation of neutralizing autoantibodies to these inflammatory mediators that are likely to participate in the regulation of cancer and autoimmunity (Figure 1).

Table 1 summarizes the appearance and role of the above anti-cytokine and anti-chemokine autoantibodies in autoimmunity, cancer, and infectious diseases.

**Table 1:** The role of autoantibodies to cytokines and chemokines in the pathogenesis of autoimmune, cancer, and infectious diseases.

Autoantibodies	Disease	Suggested role in the regulation of disease	Reference
Anti-CCL2	Prostate cancer	Restrain by inhibiting CCR2+ tumor-associated macrophages accumulation at the tumor site	(20)
Anti-TNFα	Rheumatoid arthritis, Psoriasis	Restrain by blocking TNFα	(7, 34)
Anti-CCL3	Type-1 diabetes	Restrain by blocking CCL3	(48)
Anti-IFN-γ	Type-1 diabetes	Restrain by blocking IFN-γ	(9)
Anti-IFN-α	Psoriasis	Restrain by blocking IFN-α	(34)
Anti-IL-17	Chronic mucocutaneous candidiasis (CMC)	Aggravate by blocking IL-17	(61,62)
Anti-IL-22	CMC	Aggravate by blocking IL-22	(61)

*The table summarizes data that are presented and discussed along the manuscript*.

## The Translational Aspects of These Findings

We have shown in experimental models that autoantibody production to key inflammatory cytokines/chemokines that is developed during autoimmunity and cancer diseases could be amplified by specific targeted DNA plasmids, and that this could be beneficial for the host (7, 13, 17, 20). Thus, a potential direct therapeutic implication of these studies is the use of plasmid DNA vaccines encoding key inflammatory cytokines/chemokines to which patients display autoantibody response as method to amplify each response and by so doing suppress an ongoing disease. Even though at first glance this approach looks straightforward and promising it holds two major obstacles: the first refers to the ability of plasmid DNA vaccines in human to effectively induce immune response against the gene products they encode. Previous attempts to vaccinate against viruses using plasmid DNA vaccines, even though showed some promising results in animal models (58, 59) could not be successfully extended in human. The other obstacle is the limited ability to control this antibody response once being amplified.

Another translational aspect of these findings is their use for diagnosis of diseases, in particular early diagnosis. We observed in animal models that anti-cytokine/chemokine antibody response is initiated prior to the onset of the autoimmune condition (7). The relevance of these findings in human diseases has yet to be explored. A major limitation in this approach, however, is the specificity of this response. It is hard to believe that an anti-cytokine/chemokine antibody response would be entirely disease specific. A possible way to circumvent this obstacle could by integrating this biomarker with others that could be relevant for a given disease. For example, diagnosis of cancer of the prostate that would include combination of several biomarkers, none of which is highly selective and specific. The most abundant one is the measurement of increased level of blood prostate-specific antigen, but it also has its limitations (60). It is possible that including anti-CCL2 antibody titer, in combination with other biomarkers would assist the identification of high-risk subjects for the development of disease.

Finally, the third translational approach includes the use of B cells from patients that display a significant titer of high-affinity antibodies to given cytokines/chemokines as potential source for the development of human monoclonal antibodies for therapy.

## Autoantibodies to Cytokines and Infectious Diseases

Autoimmune regulator-deficient human and mice were studied for the appearance of autoantibodies to cytokines and chemokines, and if these antibodies may affect the development of infectious diseases. The most significant observations refer to the appearance of autoantibodies to IL-17 or IL-22 and increase susceptibility to chronic mucocutaneous candidiasis (CMC) (61, 62), as these cytokines have a major role in protecting against CMC.

## Conclusion

In various cancer and autoimmune diseases, patients display selective breakdown of B cell tolerance that is likely to be T-dependent, and results in the generation of high-affinity antibody response to key inflammatory cytokines/chemokines that are predominantly expressed at the autoimmune/cancer site. Even though the underlying mechanism of tolerance breakdown is not fully understood; in some diseases, it is beneficial for the host. The translational implications of these findings may include novel therapeutics, diagnostic, and monoclonal antibodies development strategies.

## Author Contributions

The author confirms being the sole contributor of this work and approved it for publication.

## Conflict of Interest Statement

The author declares that the research was conducted in the absence of any commercial or financial relationships that could be construed as a potential conflict of interest.
